# Supplementary material for: Radioactive isotopes reveal a non sluggish kinetics of grain boundary diffusion in high entropy alloys
Source: Sci Rep. 2017 Sep 25;7:12293. doi: 10.1038/s41598-017-12551-9 (PMC5612997; doi:10.1038/s41598-017-12551-9)
Supplement: Supplementary file 1 — Supplementary information [file 41598_2017_12551_MOESM1_ESM.pdf]

Radioactive isotopes reveal a non sluggish kinetics of grain boundary diffusion in high entropy alloys

M. Vaidya[a,b], K.G. Pradeep[c], B.S. Murty[b], G. Wilde[a,d], S.V. Divinski[a,e]

[a]*Institute of Materials Physics, University of Münster,*

Wilhelm-Klemm-Str. 10, 48149 Münster, Germany

[b]*Department of Metallurgical & Materials Engineering, Indian Institute of Technology Madras, Chennai - 600036, India*

[c]*Materials Chemistry, RWTH Aachen University, Kopernikusstr.10, 52074, Aachen, Germany*

[d] *Nanjing University of Science & Technology, Herbert Gleiter Institute of Nanoscience, Nanjing 210094, Jiangsu, P. R. China*

[e]*Samara National Research University, Moskovskoye Shosse 34, Samara, 443086, Russia*

Supplementary information

The transition BC kinetic regime

Szabo *et al.* [S1] have shown that if a penetration profile measured in the transition BC kinetic regime is nevertheless analyzed according to the B- or C-type of the kinetic regimes, the determined values of P or D_{gb}, respectively, will be underestimated. The authors suggested a simple procedure to correct the experimental values and we will use it here as described in Ref. [S2]:

Taking into account that the value of α is almost unity in this experiment at 723 K, the following steps are taken:

1. The concentration profile is re-plotted against the reduced penetration depth $\alpha w^{4/5}$, where $w = \frac{x}{\sqrt{\delta D_{gb}}}\left(\frac{4D_v}{t}\right)^{1/4}$. The evaluated value of D_{gb} is used here and y is the penetration depth.
2. Then the average value of this parameter $\alpha w^{4/5}$ is determined and the correction factor, $D_{gb}^{exp}/D_{gb}^{theor}$, could be found from the graphical dependence shown in Ref. [S2]. Here D_{gb}^{theor} is the true value of the GB diffusion coefficient.

Having applied this procedure, the correction factor is found to be 0.45 and the "true" value of the Ni GB diffusion coefficient at 723 K is then 1.22×10^{-16} m^2/s.

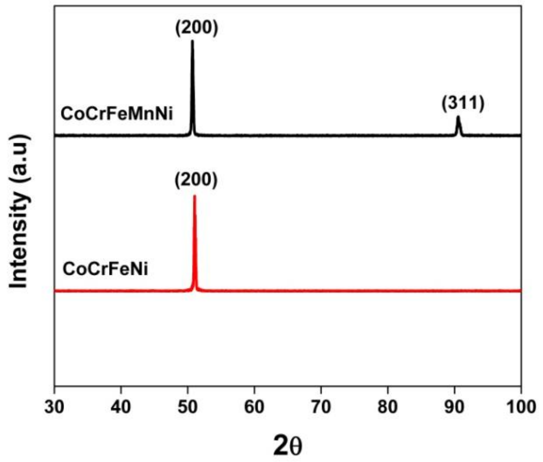

Fig S1. XRD patterns of as-homogenized CoCrFeNi and CoCrFeMnNi alloys. Only diffraction maxima corresponding to an FCC lattice are observed.

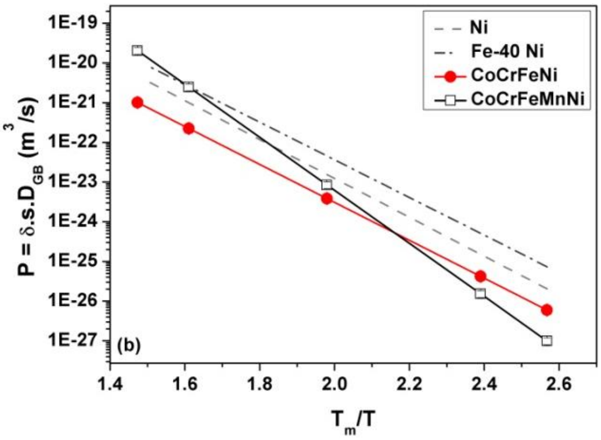

Fig S2. Arrhenius plots for Ni GB diffusion in CoCrFeNi and CoCrFeMnNi alloys and comparison of GB diffusivities (triple products) of Ni in pure Ni [27] and in Fe-Ni alloy [32] against the inverse of the homologous temperature.

Table S1: Parameters of GB diffusion measurements. The uncertainties of the determined values of P and D_{gb} do not exceed 20%.

**CoCrFeNi**							
**T (K)**	**t (10^5 s)**	**$\sqrt{D_v t}$ (nm)**	**D_{gb} (m^2s^{-1})**	**P (m^3s^{-1})**	**α**	**β**	**Kinetic regime**
673	5.78	0.1	9.6×10^{-18}	----	2.4	----	C
723	1.76	0.3	6.35×10^{-17}[a]	4.5×10^{-26}[a]	0.9	1.6×10^{8}	Transition BC
873	6.05	21	----	2.6×10^{-24}	1.2×10^{-2}	8.2×10^{4}	B
1073	11.8	808	----	1.5×10^{-22}	2.1×10^{-4}	1.3×10^{3}	B
1173	4.03	1617	----	1.5×10^{-21}	1.5×10^{-4}	7.7×10^{1}	B
**CoCrFeMnNi**							
673	5.78	0.03	5.7×10^{-18}	----	8.2	4.0×10^{9}	C
723	1.76	0.11	1.3×10^{-17}	----	2.3	2.4×10^{8}	C
873	6.05	15	----	4.0×10^{-24}	1.6×10^{-2}	1.8×10^{5}	B
1073	29.3	1698	----	3.6×10^{-21}	1.5×10^{-4}	1.1×10^{3}	B
1173	22.5	6364	----	2.6×10^{-20}	3.9×10^{-5}	1.1×10^{2}	B

[a] These are uncorrected values. For the corrected ones, see the text.

References

[S1] I.A. Szabó, D.L. Beke, F.J. Kedves, On the transition between the C and B kinetic regimes for grain-boundary diffusion, Philos. Mag. A. 62 (1990) 227–239.

[S2] A. Paul, T. Laurila, V. Vuorinen, S.V. Divinski, Thermodynamics, diffusion and the Kirkendall effect in solids, Springer, 2014.
